# Design and performance of double-layered artificial chordae

**DOI:** 10.1093/rb/rbae076

**Published:** 2024-06-27

**Authors:** Tingchao Zhang, Yichen Dou, Yang Li, Rifang Luo, Li Yang, Weiwei Zhang, Yunbing Wang, Xingdong Zhang

**Affiliations:** National Engineering Research Center for Biomaterials and College of Biomedical Engineering, Sichuan University, Chengdu, 610065, China; Hangzhou Valgen Medtech Co, Ltd, Hangzhou, 310052, China; National Engineering Research Center for Biomaterials and College of Biomedical Engineering, Sichuan University, Chengdu, 610065, China; Hangzhou Valgen Medtech Co, Ltd, Hangzhou, 310052, China; National Engineering Research Center for Biomaterials and College of Biomedical Engineering, Sichuan University, Chengdu, 610065, China; National Engineering Research Center for Biomaterials and College of Biomedical Engineering, Sichuan University, Chengdu, 610065, China; Hangzhou Valgen Medtech Co, Ltd, Hangzhou, 310052, China; National Engineering Research Center for Biomaterials and College of Biomedical Engineering, Sichuan University, Chengdu, 610065, China; National Engineering Research Center for Biomaterials and College of Biomedical Engineering, Sichuan University, Chengdu, 610065, China

**Keywords:** artificial chordae, double-layered artificial chordae, ePTFE, UHMWPE, antithrombotic, enhanced fatigue resistance

## Abstract

Surgical repair with artificial chordae replacement has emerged as a standard treatment for mitral regurgitation. Expanded polytetrafluoroethylene (ePTFE) sutures are commonly employed as artificial chordae; however, they have certain limitations, such as potential long-term rupture and undesired material/tissue response. This study introduces a novel approach to artificial chordae design, termed the New Artificial Chordae (NAC), which incorporates a double-layered structure. The NAC comprises a multi-strand braided core composed of ultra-high molecular weight polyethylene (UHMWPE) fibers as the inner core, and an outer tube made of hydrophobic porous ePTFE. Compared to traditional ePTFE sutures, NAC exhibits increased flexibility, enhanced tensile strength, longer elongation and improved fatigue resistance. Moreover, NAC exhibits a more hydrophobic surface, which contributes to enhanced hemocompatibility. The study also includes *in vivo* investigations conducted on animal models to evaluate the biocompatibility and functional efficacy of the artificial chordae. These experiments demonstrate the enhanced durability and biocompatibility of the NAC, characterized by improved mechanical strength, minimal tissue response and reduced thrombus formation. These findings suggest the potential application of NAC as a prosthetic chordae replacement, offering promising prospects to address the limitations associated with current artificial chordae materials and providing novel ideas and approaches for the development of sustainable and biocompatible regenerative biomaterials.

## Graphical Abstract

**Figure:**
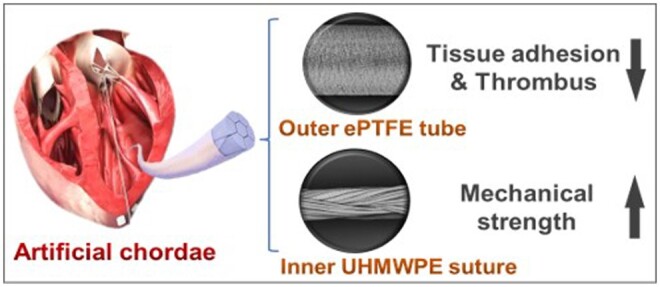

## Introduction

Mitral regurgitation (MR) represents the most prevalent heart valve disease. The use of artificial chordae as a substitute for elongated or ruptured chordae, which lead to mitral valve prolapse or regurgitation, has become the standard treatment for MR. Surgical techniques employing artificial materials for reconstructing mitral valve chordae can be traced back to the 1960s. Initially, various suture materials were proposed for replacing diseased mitral valve chordae, but their clinical applications lacked consistency. Frater *et al.* introduced the use of autologous and bovine pericardium as replacements for elongated or ruptured mitral and tricuspid valve chordae tendineae in both experimental and clinical settings [1, 2]. Although the feasibility of using autologous or allograft pericardium as artificial chordae was demonstrated to some extent, concerns regarding long-term performance and the need for more durable materials became evident [3–6].

Early cases of graft utilization consistently exhibited thickening, fibrosis and atrophy, indicating the necessity for more suitable materials to create new chordae [3]. Synthetic materials for artificial chordae have seen increasing utilization and have shown promising results in mitral valve repair surgeries, characterized by excellent durability and biocompatibility. Common materials utilized for artificial chordae include polytetrafluoroethylene (PTFE), silk, nylon, autologous tissue strips and glutaraldehyde-fixed bovine pericardium [2, 4–7]. Expanded polytetrafluoroethylene (ePTFE), a thermoplastic polymer, possesses remarkable physical, chemical, mechanical and thermal characteristics, making it widely applicable. It stands out for its flexibility, high tensile strength and fatigue resistance [8]. The suture material derived from ePTFE is a monofilament with microporous properties that allow for extensive ingrowth and infiltration of host tissue within the interstitial space [9, 10]. It also exhibits good biocompatibility and carries negative surface charges consistent with endothelial cells, reducing the likelihood of thrombosis [11, 12].

Chordal replacement using ePTFE sutures (GORE-TEX sutures; W.L. Gore & Associates, Inc, Flagstaff, Ariz) was first introduced experimentally by Frater and colleagues in the early 1980s [13]. Since then, artificial chordae tendineae made from PTFE sutures have been clinically validated [14, 15] and have become the most commonly used materials in mitral valve repair surgeries. Clinical applications have demonstrated that ePTFE sutures can yield positive long-term outcomes by maintaining normal valve function and reducing the risk of regurgitation [16–21]. However, cases of chordae failure leading to recurrent MR still occur [15, 22]. Despite evidence suggesting that even after 10 years, ePTFE chordae remain flexible and pliable, or exhibit extensive fibrosis that renders them indistinguishable from native chordae [13], there is also evidence that artificial chordae may degenerate, calcify and eventually rupture over time (refer to Supplementary Table S1) [22].

Excessive suture tension, friction, calcification, and surgical damage are common causes of ePTFE rupture [22–25]. When the mechanical stress from cardiac motion and blood flow exceeds the capacity of ePTFE sutures, it can lead to chordae tendineae rupture during the postoperative period. Additionally, fibrous reactions over time can result in dystrophic calcification, weakening the ePTFE and causing chordal failure [26]. These factors, either individually or in combination, increase the risk of ePTFE suture rupture. Therefore, further study and refinement of ePTFE-based sutures in mitral valve repair are necessary in certain circumstances. Based on clinical experiences, the ideal artificial chordae tendineae material should possess certain characteristics: (i) sufficient strength and durability to withstand mechanical stress, (ii) a certain degree of elasticity and flexibility to mimic natural chordae tendineae, enabling adaptation to heart movement and deformation, (iii) mild tissue response to ensure integration with surrounding tissues and (iv) good hemocompatibility to prevent thrombus mineral deposition that can lead to tendon stiffening and damage. Considering these characteristics comprehensively can aid in the selection and development of superior artificial chordae tendineae materials, thereby improving the effectiveness and durability of mitral valve repair surgeries.

**Figure 1.**
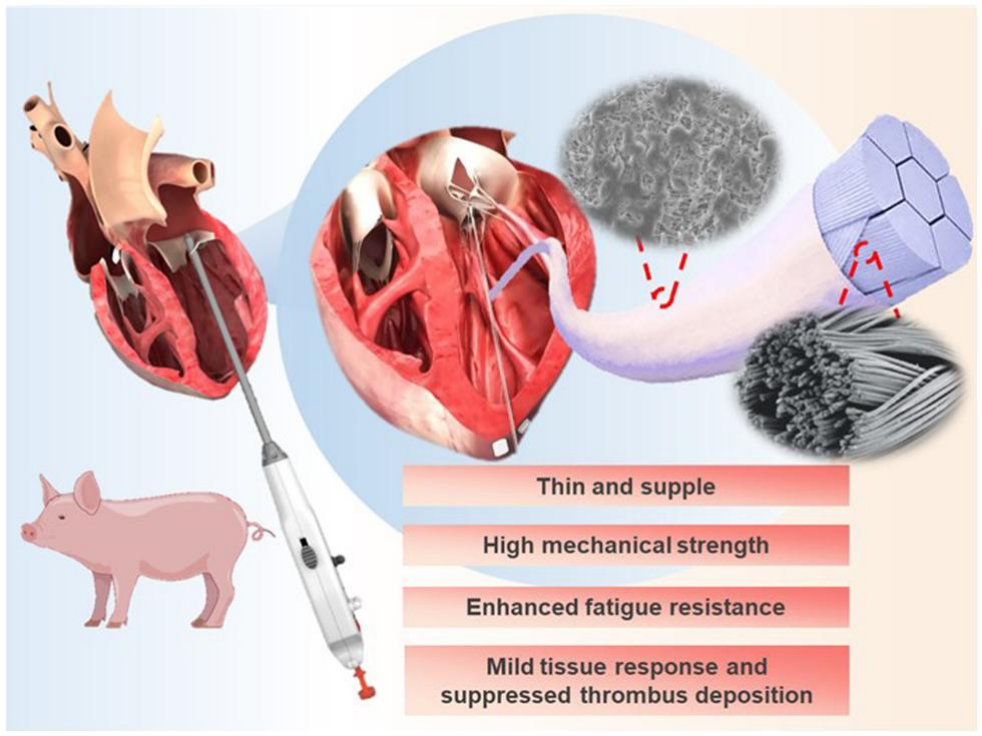
Schematic diagram of the NAC. (Image from https://www.valgenmed.com, with permission of Hangzhou Valgen Medtech Co., Ltd.)

Based on above considerations, in this study, New Artificial Chordae (NAC) with a double-layered design based on ePTFE sutures was developed to achieve superior artificial tendon material. The double-layered artificial chordae material consists of two parts, an inner core made of multi-stranded braided ultra-high molecular weight polyethylene (UHMWPE) and an outer tube made of ePTFE (Figure 1). UHMWPE fibers are known for their excellent mechanical properties and biocompatibility, commonly used in the medical field for orthopedic and soft tissue repair [27–30]. This makes them suitable for enhancing the overall strength of artificial tendons, enabling them to withstand mechanical stresses generated by cardiac motion and blood flow, thereby reducing the risk of rupture. The outer ePTFE tube is more hydrophobic and possesses anti-adhesive properties, which may induce a mild tissue response and reduce thrombus deposition, ultimately reducing the risk of adverse complications. This study conducted laboratory testing and designed animal model tests to evaluate the performance of the new double-layered artificial tendon. The results provide preliminary evidence supporting the effectiveness and feasibility of this design, thus encouraging further research and development of artificial tendon materials. The NAC is expected to improve the durability, wear resistance and compatibility with surrounding tissues of artificial tendons, ultimately enhancing their potential effectiveness and rehabilitation outcomes.

## Materials and methods

### Double-layered artificial chordae design

**Figure 2.**
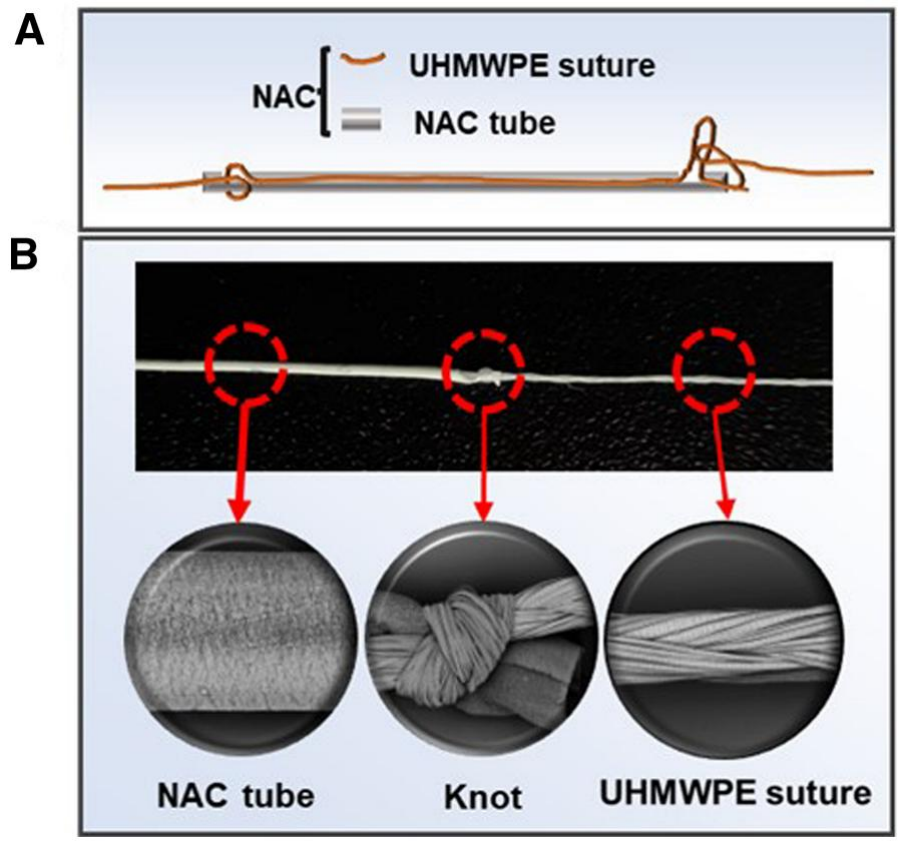
Composition and preparation process of NAC.

The newly proposed double-layered artificial chordae (NAC) design aimed to overcome the limitations of traditional ePTFE sutures. The NAC consists of two parts: the outer NAC tube (manufactured in Hangzhou Valgen Medtech Co, Ltd, Hangzhou, China) and the inner UHMWPE suture (Teleflex medical, USA). The manufacturing process involved the sintering of an ePTFE membrane (Cobetter, China) to create the NAC tube, which was then wrapped around the UHMWPE suture and secured with knots at both ends. Specifically, the ePTFE membrane, with a thickness of approximately 10 μm, a width of about 30 cm and a length of roughly 130 mm, was carefully wrapped around the mandrel, which had an outer diameter of approximately 0.3 mm, for a minimum of 10 turns, ensuring the avoidance of wrinkles during the wrapping process. Subsequently, the wrapped ePTFE membrane was securely enclosed with aluminum foil, and then inserted into stainless steel tubes with an inner diameter of 6.5 mm. Both ends were sealed with aluminum foil, and the assembly was placed into an electrically heated circulating air chamber furnace for heat setting, under conditions of 350°C–400°C for a duration of 3–5 min. After heat setting, the assembly was allowed to cool naturally. The mandrel was subsequently removed, and grooves were processed at both ends of the tube. The UHMWPE suture was passed through the center of the NAC tube, and knots were tied at the respective grooves within the tube, ensuring the fixation of both the UHMWPE suture and the NAC tube. This marked the completion of the NAC production (Figure 2). The outer NAC tube provides biocompatibility as a tissue and blood contact material, while the inner UHMWPE suture contributes mechanical properties. As a control, commercially available ePTFE sutures (referred to as ePTFEs) (W.L. Gore & Associates Inc. Elkton, Md) were used in the experiments and compared to the NAC design.

### Characterization

#### Surface characteristics

The surface and cross-sectional micromorphology of NAC and ePTFEs were observed by scanning electron microscopy (SEM, JEOL, JSM-7500F, Japan). The surface chemical composites of NAC and ePTFEs were verified by ATR-FTIR (Spectrum One, Nicolet, USA) with wavelengths ranging from 500 to 4000 cm^−1^. The hydrophobicity surface contact angle was measured by contact angle meter (Biolin Scientific, Attension Theta, Switzerland). The surface roughness of the scaffolds was determined by Atomic Force Microscopy (AFM; Bruker, Dimension ICON, Germany).

#### Tensile test

The NAC and ePTFEs were cut into 20 cm, and then the samples were mounted on the tensile tester (Bio-Tester 5000, Cellscale, UTM4103). The samples were stretched at a constant velocity of 30 mm/min. Setting the scale distance to 3 mm, and the maximum load applied in the experiment was 0.75 N. Record the tensile curve and maximum breaking force, and calculate the tensile strength, Young’s modulus and elongation at break of samples based on the stress-strain curve.

#### Fatigue test

In order to simulate the force pattern of artificial tendon cords in the human body, ePTFEs and NAC were placed in PBS buffer (Invitrogen, USA) at 37°C, and accelerated fatigue experiments were performed on a fatigue testing machine (TA, USA, ElectroForce 3500) at a frequency of 30 Hz. After reaching the set time, the samples were removed for observation and tested for tensile strength.

### Hemocompatibility

#### Ethical issues on animal experiments

All animal experimental procedures were conducted in accordance with the Guidelines for Care and Use of Laboratory Animals of Sichuan University and approved by the Medical Ethics Committee of Sichuan University (approval No. KS2002866).

#### Hemolysis rate

Red blood cells (RBCs) from rabbits were collected by centrifugation at 1500 rpm for 10 min. The RBCs were then diluted 5-fold with PBS pH 7.2 (Gibco). The various samples (cut into 1.5 cm pieces) were then gently immersed in 1.5 ml of RBC dispersion and incubated for 2 h. RBCs treated with deionized water were used as positive control and RBCs treated with PBS were used as negative control. After incubated, all the samples were centrifuged at 1500 rpm for 10 min, the supernatant was collected and diluted three times, and the absorbance was measured at 541 nm with an enzyme meter.

The hemolysis rate of erythrocytes (%) was calculated as follows:

$$\mathrm{Hemolysis}\;\mathrm{rate}\;(\%)\;=\;\frac{A_{\mathrm{sample}}-A_{\mathrm{negative}}}{A_{\mathrm{positive}}-A_{\mathrm{negative}}\;}\times 100\%.$$

There were five parallel samples in each group.

#### Platelet adhesion test

Fresh rabbit whole blood was collected using vacuum tubes containing sodium citrate (the ratio of blood to sodium citrate was 9:1). The whole blood was centrifuged at 1500 rpm for 15 min to obtain platelet-rich plasm (PRP). The samples were incubated with PRP in a shaker at 37°C for 1 h. After incubation, the samples (*n* = 6) were rinsed with sterile PBS three times to remove non-adherent platelets. The morphology of platelet adhesion and activation on sample surfaces was qualitatively observed by SEM. Platelets adhering to the samples were stained with fluorescein diacetate (FDA, Invitrogen, USA), FDA was diluted to 10 μg/ml and incubated for 3 min at room temperature, and the platelets were washed with PBS buffer to remove unbound FDA and other impurities, and then examined and visualized with a laser scanning confocal microscope (Leica SP5, Germany).

#### In vitro whole blood adhesion assay

Rabbit blood was collected as described previously and used within 1 h. NAC and ePTFE samples were prepared by cutting them into 1 cm pieces, rinsing them with saline, and placing them in a 24-well plate. Each well was filled with 300 μl of rabbit blood and incubated at 37°C with gentle shaking at 70 bpm for 1 h. Following the incubation, the blood was discarded, and 500 μl of saline was added to each well to rinse off any unadhered whole blood through gentle plate shaking. The adherent blood cells were immobilized by submerging the cleaned samples in a 2.5% (w/w) glutaraldehyde solution (Servicebio, China) for 4 h. Subsequently, the samples were dehydrated using graded concentrations of ethanol (25%, 50%, 75% and 100%, v/v) for 20 min at each concentration. After drying, the SEM was used to observe and capture images of the whole blood adhesion on each sample group.

#### Ex vivo antithrombogenicity assay

Male New Zealand white rabbits weighing between 3 and 3.5 kg (*n* = 3) were selected for the study. The rabbits were injected with pentobarbital sodium for anesthesia. Prior to the experiment, the experimental tubes were soaked overnight in a heparin solution. The NAC and ePTFE samples were carefully fixed to the inner wall of the tubes, and the entire surface was moistened with normal saline (NS). The left carotid artery and right jugular vein of the rabbits were meticulously isolated and connected to the assembled tubes, creating an arteriovenous extracorporeal circuit. After being subjected to extracorporeal circulation for 40 min, the materials were removed, washed with NS, photographed and subsequently fixed overnight using paraformaldehyde. Following fixation and dehydration, the micromorphology of the samples was observed using SEM.

#### Adsorption of proteins

The FITC-BSA solution (Solarbio, China) was prepared by diluting it to a concentration of 1 mg/ml in PBS under dark conditions. Prior to adding the FITC-BSA solution, the NAC, ePTFEs, NAC tube and ePTFE film samples were rinsed multiple times with PBS. The samples were then incubated with the FITC-BSA solution in the dark for 1 h. After the incubation, each sample was rinsed several times with PBS to remove any unbound proteins. The protein adhesion on the samples was quantitatively or qualitatively assessed using a fluorescence microscope and Image J software.

### Cytocompatibility

#### Cytotoxicity

ZDBC polyurethane film (HRI/FDSC, Japan) (6 cm^2^/ml, shaken at 37°C for 24 h) was used as the positive control, and high-density polyethylene film (HRI/FDSC, Japan) (3 cm^2^/ml, shaken at 37°C for 24 h) was used as the negative control. The test samples, positive control and negative control extracts were mixed with mouse fibroblast L929 to form a cell suspension at a concentration of 1 × 10^5^/ml, and inoculated into 96-well plates with 100 μl in each well. All plates were incubated at 37°C for 24 h under 5% (v/v) carbon dioxide. Discard the original medium, add 50 μl of 1 mg/ml MTT (Sigma, USA) solution to each well, and incubate at 37°C for 2 h. then discard the MTT solution, 100 μl isopropanol (CNW, China) was added to each well and mixed for 10 min on an oscillator. Detect the OD value of each well at 570 nm using an enzyme marker and calculate the cell survival rate of each group according to the following formula:

$$\mathrm{Cell}\;\mathrm{viability}\;\%\;=\;\frac{{\mathrm{OD}}_{570}}{{\mathrm{OD}}_{570b}}$$

In the formula, OD_570_: average optical density of 100% extraction of test samples; OD_570b_: mean value of blank optical density.

#### Subcutaneous implantation assay

Prior to this experiment, the samples were immersed in 75% alcohol for 3 h and then exposed to ultraviolet light overnight for sterilization. First, male Sprague Dawley rats (*n* = 6, weighing 150 ± 10 g) were anesthetized with pentobarbital, then the dorsal skin of SD rats was surgically peeled and NAC and ePTFEs were implanted into subcutaneous pockets (two pockets on the back of each rat). After 7 and 14 days of implantation, the specimens with the surrounding capsule were removed from the rats and fixed in 10% paraformaldehyde to evaluate the degree of inflammation.

#### Histological analysis

As for the histological analysis, the samples with surrounding tissues were collected to evaluate the tissue response. The tissues were fixed with paraformaldehyde and then embedded in paraffinand stained with H&E (Servicebio, China) and anti-CD 68 antibody (Servicebio, China) for further investigation. At least three random view fields were imaged for each sample, and the fibrous encapsulation thickness were quantitively analyzed by Image J software.

### Implantation of NAC in pigs

#### Animal model

Nine pigs (weighing 80–90 kg) were used as animal models to study the NAC. The animals were divided into a short-term group (three pigs, sacrificed on the 90th day after the procedure), and a long-term group (six pigs, sacrificed on the 180th day after the procedure).

#### Animal procedure

**Figure 3.**
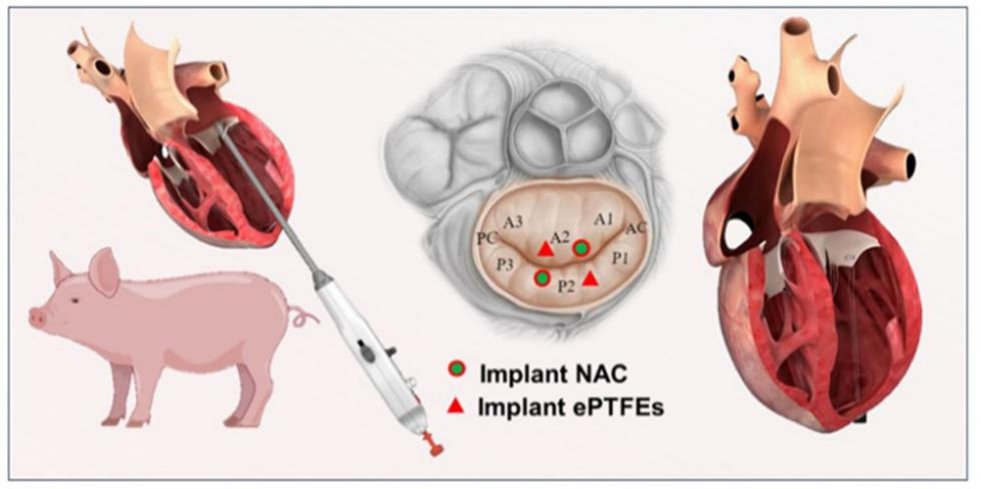
Schematic diagram of the implantation position of the artificial tendon cords in pigs.

The procedure was performed under general anesthesia with fluoroscopic and direct echocardiographic guidance. The ePTFE sutures and NAC were preloaded into MitralStitch instruments, and after exposing the heart in each animal through an open chest, as shown in Figure 3A transapical approach was used to implant a set of (one set equaled two sutures) ePTFE sutures and NAC sutures into the anterior and posterior mitral leaflets of the mitral valve, respectively, the length of each suture is 8 cm. under ultrasound guidance, and the four sets of prosthetic chords were fixed in the apices of the heart. Follow-up examinations were performed at 90 and 180 days postoperatively.

#### Evaluation protocol

Echocardiographic evaluation was performed immediately after, and on the 90th and 180th days after the procedure. Animals were euthanized according to the manufacturer's instructions. Artificial cords placement, tissue injury and thrombosis were evaluated after the left atrium and left ventricle were opened. At the same time, the heart, liver, spleen, lung, kidney and brain were also collected for gross examination, and pathological sections were utilized to look for thromboembolism and tissue changes.

### Statistical analysis

Each experiment in this work was replicated no less than three times in parallel, and the results were provided as mean ± standard deviation. Significant differences between groups under the same test were determined using ANOVA. The threshold for statistically significant was established as a probability value (*P*) below 0.05. For example, the values *P* < 0.05, *P* < 0.01, *P* < 0.001, and *P* < 0.0001 would be represented by the symbols *, **, *** and ****.

## Results

### Characterization of NAC and ePTFE sutures

**Figure 4.**
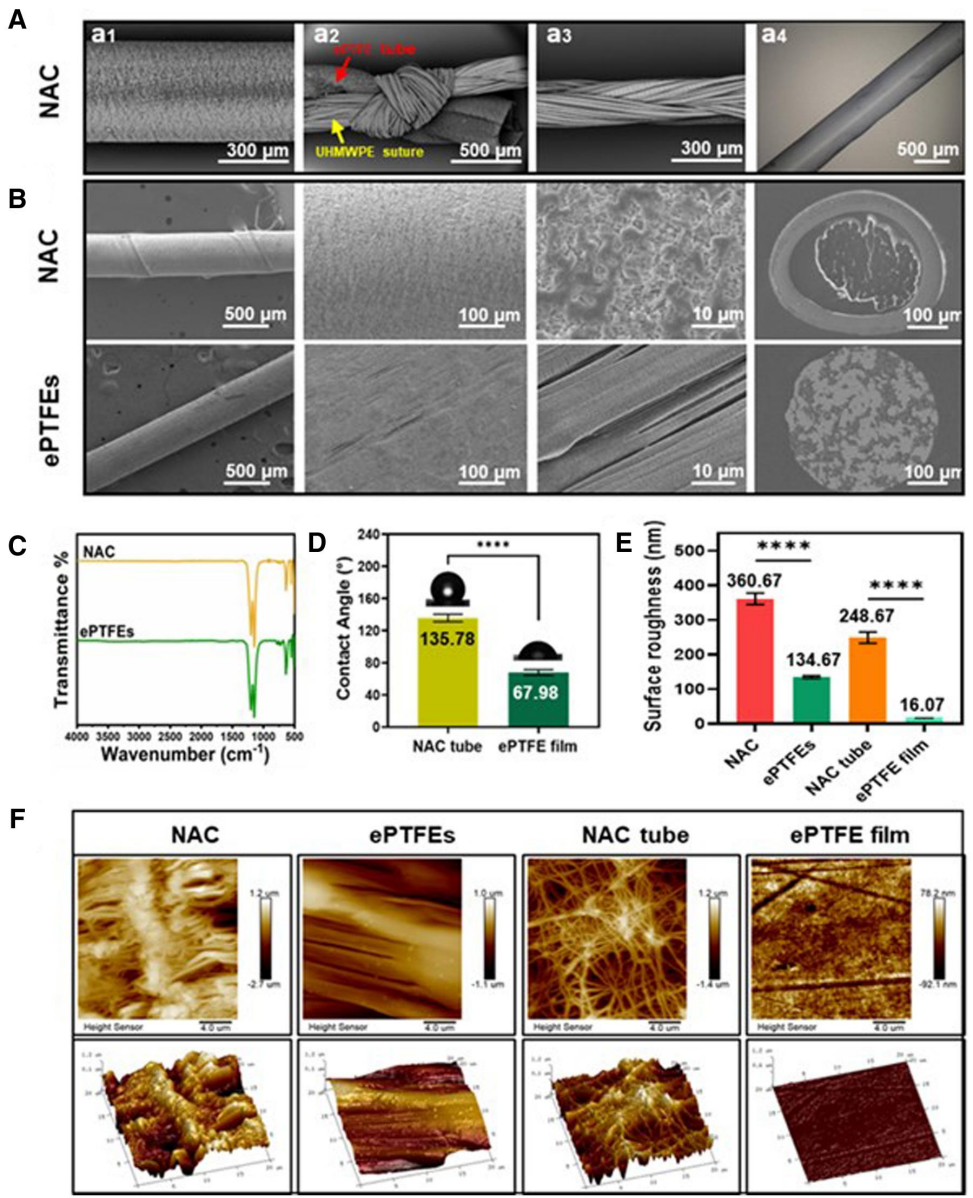
(**A**) Structure of the NAC. (**B**) Surface and cross-section morphology of NAC and ePTFEs. (**C**) ATR-FTIR spectroscopy of NAC and ePTFEs. (**D**) Contact angle of NAC tube and ePTFE film. (**E**) Surface roughness and AFM images (**F**) of different materials (*n* = 3). Data are presented as mean ± SD and analyzed using one-way ANOVA and unpaired *t*-test. Note: **** *P* < 0.0001.

Figure 4A showed the structure of the NAC. The porous NAC tubes (Figure 4A1), the knots (Figure 4A2), and the inner multi-strand braided UHMEPE sutures (Figure 4A3) were used to make up the NAC, which was constructed by knotting the ends in such a way that it was structurally stable and cannot be easily separated from the inner and outer layers. The bilayer structure of NAC can be seen through a light microscope (Keyenc, Japan, VHX-6000) (Figure 4A4). The surface and cross-sectional morphology of NAC and ePTFEs were observed by SEM (Figure 4B). The surface of NAC has an irregular porous network structure, while the surface of ePTFEs has a larger pore size and was oriented and distributed along the axial direction of the suture line. The cross-section of NAC clearly shows its double layer as well as the internal multi-strand braided UHMEPE sutures, and the cross-section of ePTFEs also shows its internal porous structure.

ATR-FTIR was used to characterize chemical characterization on surfaces of NAC (that was the ePTFE tube). As shown in Figure 4C, strong absorption peaks are observed in the range of approximately 1000–1300 cm^−1^, representing the stretching vibration of C–F bonds. Weak absorption peaks may be observed in the range of approximately 1150–1250 cm^−1^, representing the stretching vibration of C–CF_2_ bonds. Additionally, weak absorption peaks may be observed in the range of approximately 1250–1450 cm^−1^, representing the bending vibration of CH_2_ groups. Strong absorption peaks can be observed in the range of approximately 600–900 cm^−1^, representing the bending vibration of CF_2_ groups in ePTFE [27–29]. From the figure, it can be seen that the above characteristic peaks of NAC and ePTFEs overlap, indicating that the heat treatment did not destroy the chemical structure of the outer ePTFE tubes during the processing and manufacturing of NAC, but only changed the surface morphology on the physical layer.

Surface hydrophilicity plays a significant role in directing material/tissue response. For example, the adhesion of proteins and cells may prong to happen on hydrophilic surfaces. Water contact angle experiments were performed using unfolded flat unprocessed NAC tube (Cobetter, China) and the commercially available ePTFE film with a dense and non-porous surface (Zeus Company LLC, USA) was used as the control group. Figure 4D shows that the contact angle of the NAC surface (mean value = 135.87°) was much higher than that of the commercial ePTFE film (mean value = 67.98°) which shows significantly increased hydrophobicity, which may help to improve water-resistance behavior [30, 31].

The surface roughness of different materials was measured by AFM. The roughness increased in the following order: NAC > ePTFEs, NAC tube > ePTFE film (Figure 4E). The roughness of the NAC tube increases when it is thermally processed and coiled into the NAC suture (Figure 4F). The surface roughness of hydrophobic materials can affect the contact angle. For hydrophobic materials with contact angles >90°, according to the Cassie-Baxter model, when a liquid fills between the concave and convex structures on the rough surface of a hydrophobic material, a portion of the liquid forms a composite interface with the gas, and this composite interface results in a larger contact angle [32, 33]. Noteworthily, in this study, it is not easy to evaluate the water contact angle of the NAC tube and ePTFE suture. Research has revealed that if the surface of a material is hydrophobic, the rougher morphology may usually lead to a more hydrophobic surface [34, 35]. As shown in Figure 4D and E, we can speculate that the final product NAC and ePTFE suture may be more hydrophobic (water contact angle >135.87° and 67.98°, respectively), as they hold a larger surface roughness compared with NAC tube and ePTFE film, indicating increased water-repulsing ability.

### Mechanical properties of NAC

The mitral chord connects the mitral valve leaflets to the ventricular myocardium. If the prosthetic chord becomes overstretched, loose, or even torn, and is unable to provide sufficient tension to keep the mitral valve properly closed, blood may flow back into the left atrium during systole of the heart, which can lead to increased cardiac stress, decreased heart function, and other cardiovascular problems [13, 36]. Therefore, the artificial tendon cable should be strong and stable enough to ensure that the mitral valve functions properly during systole and diastole and to prevent regurgitation [37].

**Figure 5.**
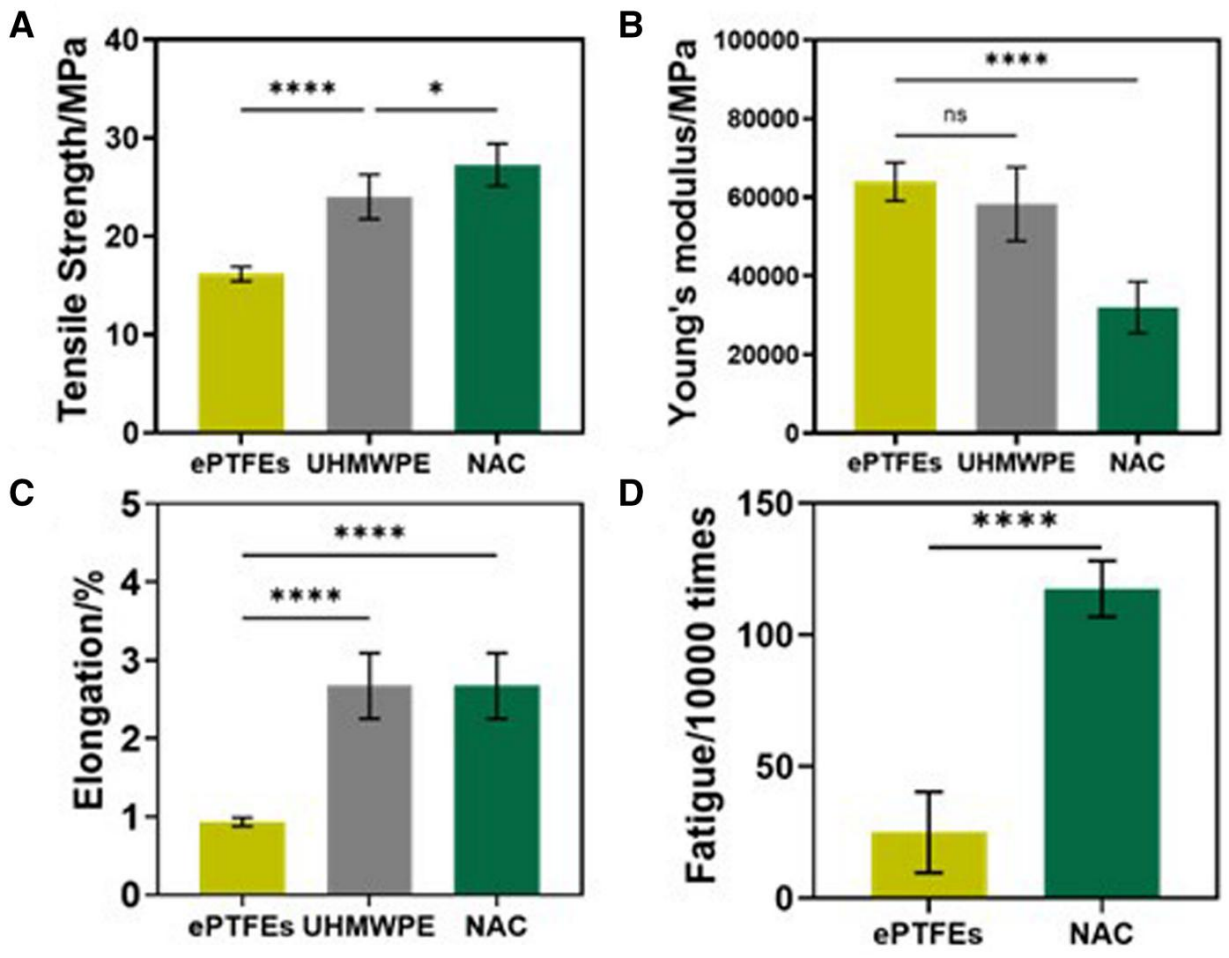
Mechanical property of different samples. (**A**) Tensile strength (*n* = 5), (**B**) Young’s modulus (*n* = 5), (**C**) elongation (*n* = 5) and (**D**) fatigue (*n* = 4). Data are presented as mean ± SD and analyzed using a one-way ANOVA. Note: ns, no significance, * *P* < 0.05 and **** *P* < 0.0001.

Tensile strength, Young’s modulus, elongation and fatigue of NAC, ePTFEs and UHMWPE suture were tested to characterize mechanical properties. According to Figure 5A, the tensile strength of NAC (27.3 ± 2.2 MPa) was higher than that of ePTFEs (16.2 ± 0.8 MPa) and UHMWPE (24.0 ± 2.3 MPa), indicating that NAC can withstand greater tensile forces under the same conditions. The artificial tendon needs to withstand forces during the contraction and relaxation processes of the heart, making higher tensile strength crucial for ensuring stability and durability. The Young’s modulus of NAC was lower (32 050 ± 6526 MPa) compared to ePTFEs (63982 ± 4893 MPa) and UHMWPE (58 302 ± 9368 MPa) (Figure 5B). The Young’s modulus reflects the material’s stiffness or elasticity, with higher values indicating greater resistance to deformation [38]. Therefore, ePTFEs are relatively rigid when subjected to stress, while NAC is relatively flexible. This flexibility can be advantageous for artificial tendons as it allows for better adaptation to the heart’s movements, reducing friction and pressure on the mitral valve and surrounding tissues. Elongation at break refers to the extent to which a material can stretch during the tensile process, while elongation represents the actual amount of deformation during stretching [39]. NAC exhibits higher elongation compared to ePTFEs (Figure 5C), indicating greater extensibility under stress. This can reduce the stress on the tendon during motion, lowering the risk of damage and fatigue. In contrast, the similar elongation values between UHMWPE and NAC suggest that the excellent stretchability of NAC was primarily provided by UHMWPE.

In practical applications, tendon cords are constantly subjected to repetitive force and strain loading, making durability a key indicator for evaluating the mechanical properties of artificial tendon cords. Fatigue refers to the phenomenon where materials gradually incur damage and failure after undergoing repeated loading and unloading cycles [40]. Fatigue experiments can be conducted to assess the fatigue life of artificial tendon cord materials, which represents the number of cyclic loads the material can withstand under a certain number of cycles. This helps determine the reliability of the material and provides a basis for designing and selecting suitable artificial tendon cord materials. As shown in Figure 5D, the ePTFEs fractures after an average of 250 025 cycles, while NAC fractures only after an average of 1 175 350 cycles of continuous cycling. These results further support the superior durability of NAC material. NAC can withstand more cyclic loads without fracturing, indicating better fatigue resistance. Therefore, it was more suitable for applications such as artificial tendon cords that need to endure long-term cyclic stresses.

In summary, NAC material demonstrates superior mechanical properties when compared to ePTFEs. It exhibits higher tensile strength, lower Young’s modulus, higher elongation and better fatigue resistance. These characteristics establish NAC as a reliable and durable option, particularly suitable for applications that demand long-term resistance to forces and strains, such as artificial tendon cords. The exceptional mechanical properties of the dual-layer NAC structure are accomplished by incorporating UHMWPE as the core material. The UHMWPE core enhances strength and stiffness, contributing to the overall tensile strength and rigidity of the NAC material. Moreover, the UHMWPE core enables improved load distribution and stress transfer within the structure, ultimately enhancing its durability and fatigue resistance. By combining the unique properties of UHMWPE with the outer layer material, the dual-layer NAC structure exhibits superior mechanical performance, making it an ideal choice for high-strength and durable applications.

### Biocompatibility

#### Hemocompatibility

Thrombus formation within the artificial tendon cords may pose a risk of embolization, where fragments of the thrombus break off and travel to other parts of the body, potentially causing blockage or damage to vital organs or blood vessels. Therefore, it was critical to minimize thrombus formation and improve the blood compatibility of the artificial tendon cords to reduce the risk of embolization and ensure patient safety [41].

**Figure 6.**
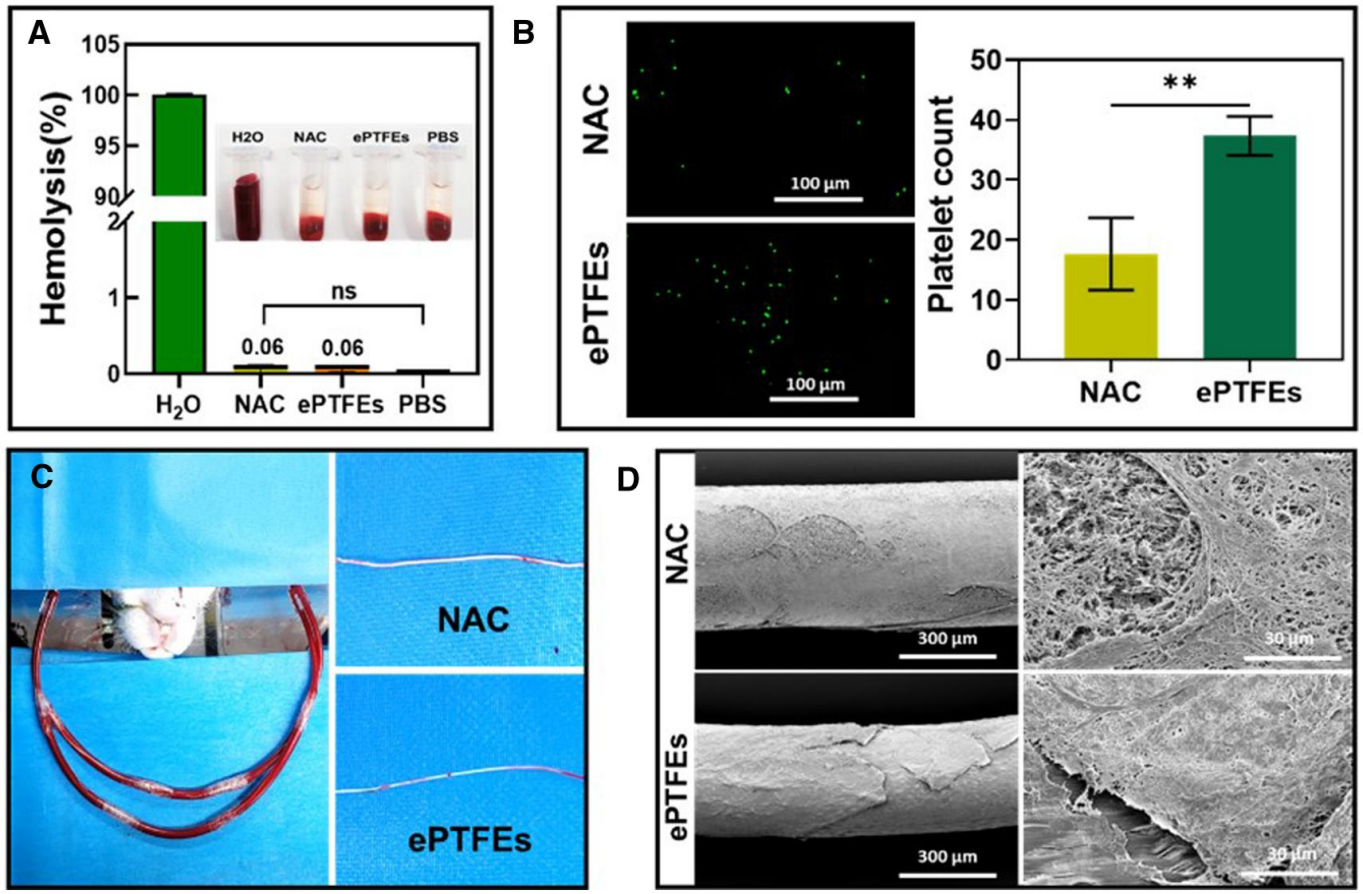
Hemocompatibility. (**A**) Photographs and quantitative results for hemolysis effect on mouse RBCs for different samples (*n* = 6). (**B**) Fluorescence images and counts quantitative results of platelets adhered on NAC and ePTFEs surfaces (*n* = 3). (**C**) Illustration of *in vitro* arteriovenous shunt assay and photographs of thrombus on the surfaces of samples after 40 min of *in vitro* blood circulation. (**D**) SEM images after 40 min *ex vivo* arteriovenous shunt assay. Data are presented as mean ± SD and analyzed using one-way ANOVA and unpaired *t*-test. Note: ns, no significance, *** *P* < 0.001.

First, the material–erythrocyte interaction was investigated using an erythrocyte hemolysis rate assay. The results of the hemolysis rate analysis in Figure 6A, showed that both NAC and frontal PTFE groups exhibited a hemolysis rate < 1%. According to ISO/TR7406, the critical safety range for hemolysis of biomaterials was considered to be within 5%, thus NAC has good hemocompatibility.

Reducing the degree of coagulation and inflammation reduces the risk of poor endothelialization and hyperplasia, thereby reducing the failure rate of cardiovascular implantable devices [42–44]. The adhesion and activation of platelets on a foreign surface generally trigger blood clotting [45]. Hemocompatibility was characterized by platelet adhesion test and *ex vivo* arteriovenous shunt assay [46]. In Figure 6B, the green fluorescent dots represent platelets adhering to the NAC and ePTFEs surfaces, and the counting and quantification results showed that relatively more green fluorescent dots were found on the ePTFEs, whereas the number of platelets adhering to the NAC was lower.

During the early stages of implantation, disordered protein adhesion and denaturation can trigger several undesirable biological responses, including thrombosis and inflammation [47–49]. Therefore, the development of an artificial tendon with a surface that reduces protein adsorption may reduce the extent of disordered responses. The protein fluorescence intensity of NAC and ePTFEs was reported as (184.56 a.u.) and (195.11 a.u.), respectively (Supplementary Figure S1). During the fabrication, the roughness of the surface of the NAC increased compared with the NAC tube, providing highly hydrophobic properties that can reduce the retention and contact time of blood components. The more hydrophobic NAC demonstrates a stronger ability to resist protein adsorption. The lower intensity of protein adsorption fluorescence in the NAC group showed a correlation trend with the results of the contact angle experiment (Figure 2D) and surface roughness (Figure 2F). This could be attributed to the hydrophobic nature of the porous surface of NAC, due to the water-repelling ability of the hydrophobic surface, proteins tend to remain in the aqueous phase rather than adsorbing on the NAC surface [30, 31]. Thus, the hydrophobic nature of the artificial tendon surface can help to reduce the adhesion of blood components (serum protein, platelets and RBCs), thereby lowering the risk of acute thrombus formation [50, 51].

To simulate real-world contact with blood, an *in vitro* arteriovenous shunt test was performed to further evaluate the antithrombotic properties (Figure 6C). Compared to ePTFEs, NAC showed a lower thrombosis rate after 40 min of *in vitro* blood circulation. Consistent with previous results, ePTFEs formed a thicker and continuous fiber network wrapping layer on its surface, which may pose a higher risk of thrombosis. In contrast, NAC had thinner fiber wrappings on the surface (Figure 6D). These findings indicate that the NAC exhibits stronger anti-thrombotic properties, likely due to its hydrophobicity that inhibits the adsorption and activation of blood components on its surface. Therefore, the NAC presented suitable hemocompatibility for further applications as blood-contacting materials.

#### Cytocompatibility

**Figure 7.**
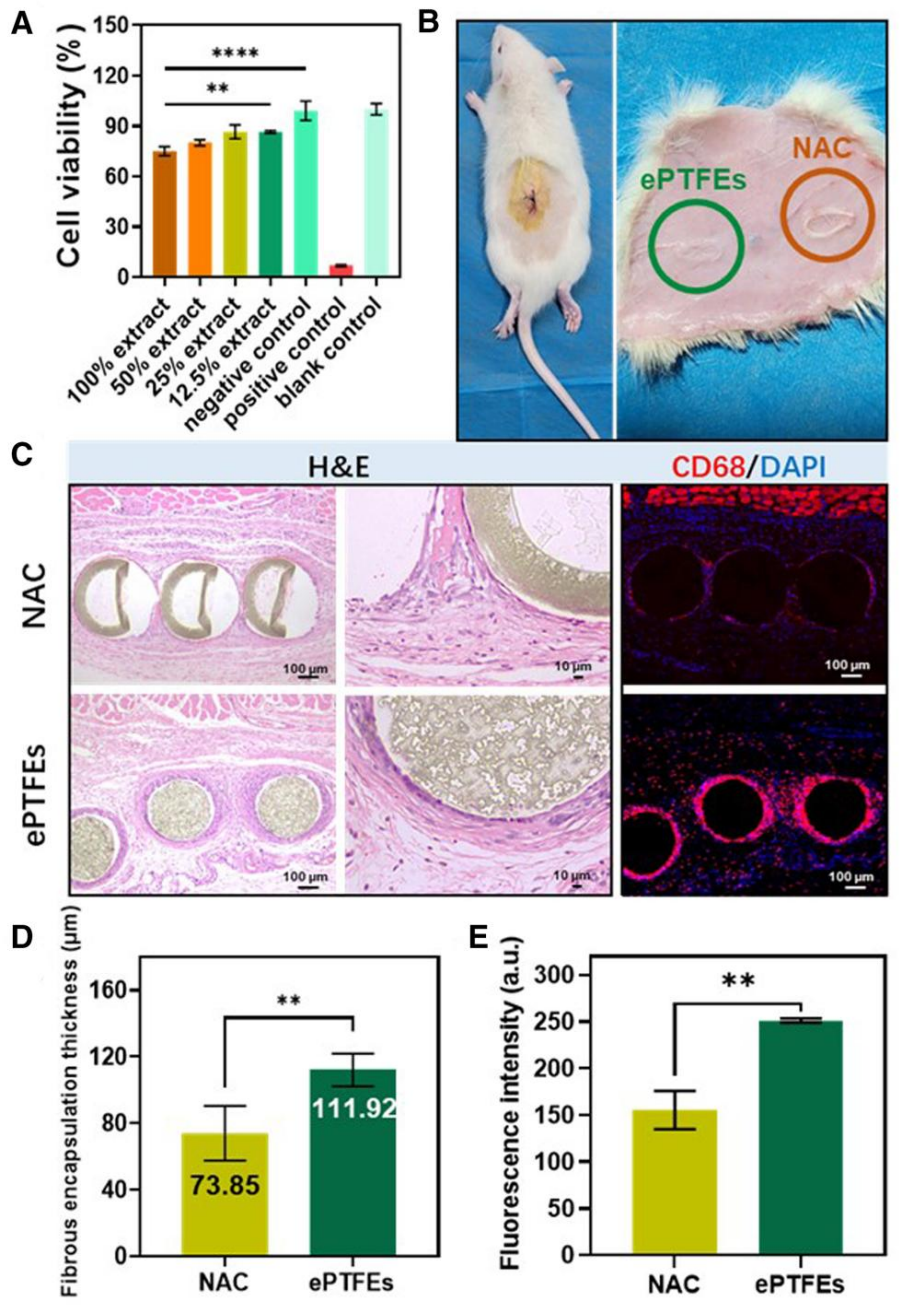
Evaluation of inflammatory response. (**A**) Cell viability of L929s in cell extracts with different concentrations after incubation for 24 h at 37°C (*n* = 3). (**B**) Schematic representation of the subcutaneous implant procedure. (**C**) H&E and CD 68/DAPI staining of subcutaneously implanted samples for 7 days. (**D**) Fibrous encapsulation thickness counted from H&E-stained graph (*n* = 7). (**E**) Statistical values of fluorescence intensity of CD 68 in different samples (*n* = 3). Data are presented as mean ± SD and analyzed using one-way ANOVA and unpaired *t*-test. Note: *** *P* = 0.0002. **P < 0.01. **** *P* < 0.0001.

Artificial tendon chordae are in direct contact with cells in the human body, so assessing their toxicity to cells was a critical step in ensuring their safety and biocompatibility, cytotoxicity assays were first performed. The cell survival rate was 75.03%, 80.09%, 86.60% and 86.57% for 100% extract, 50% extract, 25% extract and 12.5% extract of the sample group, respectively, and 6.97% for the positive control group and 99.04% for the negative control group (Figure 7A). Since the cell survival rate of the 100% extract of the test material was more than 70%, it can be judged that NAC was not cytotoxic according to the content of GB/T 16886.5-2017.

Taking these results together, it can be concluded that NAC has good biocompatibility as an artificial tendon cord material. Its surface has less adsorption of proteins, which helps to reduce the occurrence of adverse biological reactions, while it has good compatibility with cells. These properties make NAC a promising candidate material that can play an important role in the research and application of artificial tendon cords.

#### In vivo subcutaneous implantation studies

The inflammatory response around the material/tissue interface has a significant impact on the outcome of implantation. Subcutaneous implantation was a commonly used validation model to further investigate the host response *in vivo* after material implantation [52]. Macrophages adhere to the biomaterial surface after implantation and rapidly produce pro-inflammatory cytokines, reaching a peak concentration within 24 h [53]. Furthermore, the degree of cellular infiltration and the thickness of the fibrous membrane surrounding the specimens were found to be strongly correlated with inflammatory response, biocompatibility and host integration [46, 54]. In general, the higher the degree of inflammatory cell infiltration and the thicker the fibrous membrane after implantation, the more severe the host inflammatory response [55].

To directly compare the effects of the two materials on the surrounding tissues and to evaluate the acute anti-inflammatory properties of the samples. NAC and ePTFEs coils were implanted on the left and right sides of the back of each rat (Figure 7B). The samples were implanted subcutaneously in SD rats for 7 days and then removed for hematoxylin and eosin (H&E) and CD 68 staining (Figure 7C). The average thickness of the fibrous capsule and the expression intensities of CD 68 were determined (Figure 7D and E).

In the case of ePTFEs, after 7 days of implantation, thicker encapsulation was observed with an average thickness of 111.92 ± 9.82 μm more than that of NAC (73.85 ± 16.38 μm) (Figure 7D). This thicker encapsulation suggests a more pronounced tissue response around the ePTFE material. Additionally, The CD 68 staining results indicate a higher presence of inflammatory cells within the newly formed capsules around the ePTFEs. In contrast, there was a minimal presence of inflammatory cells within the capsules formed around the NAC specimens (Figure 7C and E). The findings indicate that NAC exhibits a lower inflammatory response and better tissue compatibility compared to ePTFEs. The reduced encapsulation and inflammatory cell infiltration around NAC samples suggest that NAC was more biocompatible and promoted a more favorable tissue response.

### Animal study

#### Ultrasonography results and gross anatomy

A total of nine pigs were included in the study. A 100% procedural success rate was achieved with no death, and the tendons maintained good mechanical efficacy throughout the study, no artificial chordae rupture, natural valve tissue damage or other serious procedure-related adverse events occurred. The evaluation indicators of cardiac function at different follow-up times were shown in Table 1. Electrocardiograms are detailed in Supplementary Figure S2.

**Table 1.** Echocardiographic findings

90 days	Baseline	1 M	*P-*value	3 M	*P-*value
EDV/ml	107.40 ± 11.98	108.65 ± 16.16	0.39	104.65 ± 12.62	0.76
EF/%	53.75 ± 4.83	54.60 ± 3.84	0.79	54.35 ± 9.10	0.91
ESV/ml	50.05 ± 10.02	49.73 ± 10.51	0.97	48.23 ± 14.15	0.84
FS/%	28.28 ± 3.15	28.38 ± 2.41	0.96	28.35 ± 5.91	0.88

180 days	Baseline	1 M	*P-*value	6 M	*P-*value
EDV/ml	106.83 ± 21.26	97.38 ± 18.08	0.18	138.40 ± 45.92	0.26
EF/%	63.73 ± 9.37	57.70 ± 13.79	0.50	70.98 ± 13.48	0.41
ESV/ml	39.25 ± 13.76	43.03 ± 22.45	0.78	44.28 ± 29.09	0.77
FS/%	34.80 ± 6.66	30.83 ± 8.79	0.50	41.88 ± 12.06	0.34

EDV, end-diastolic volume; EF, ejection fraction; ESV, end-systolic volume; FS, LV fractional shortening.

After the implantation of artificial tendons, the cardiac function might have been influenced by the presence of the implant, undergoing an initial adaptation period. As the postoperative time increased, the heart gradually adapted to or repaired its response to the artificial tendons. The values of EDV/ml, EF/%, ESV/ml and FS/% gradually recovered to a state closer to the pre-implantation level.

**Figure 8.**
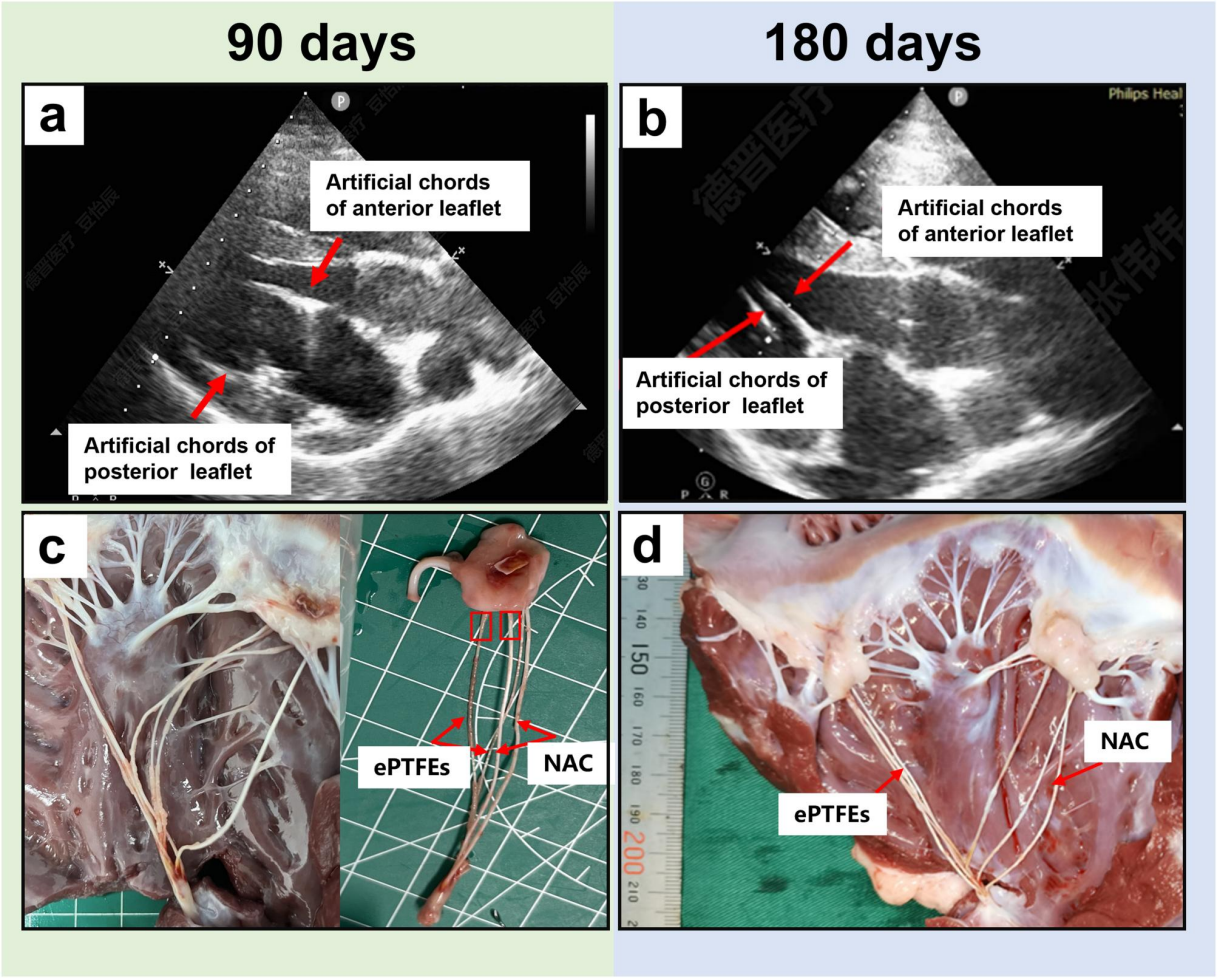
Echocardiograms at 90 (**A**) and 180 (**B**) days after surgery with the Mitralstitch system in pig model (arrows show artificial tendon cords). The gross observation of the porcine heart at 90 (**C**) and 180 (**D**) days postmortem.

The contrast of the implanted artificial tendon chordae was visualized on echocardiography (Figure 8A and B), Prior to euthanasia, transthoracic echocardiography demonstrated no or trace MR in all survivors. Neoplastic tissues were observed on the surface of all artificial tendon cords (Figure 8C and D). The surface of the ePTFE suture has localized non-endothelial-like tissue encapsulation, resulting in marked thickening of the suture, which was suspected to be a thrombus; Whereas the overall surface of the suture was smooth with only a few thrombus-like particles attached; mainly concentrated near the upper and lower anchorage of the NAC, which climbed and grew along the surface of the NAC.

#### SEM observation

**Figure 9.**
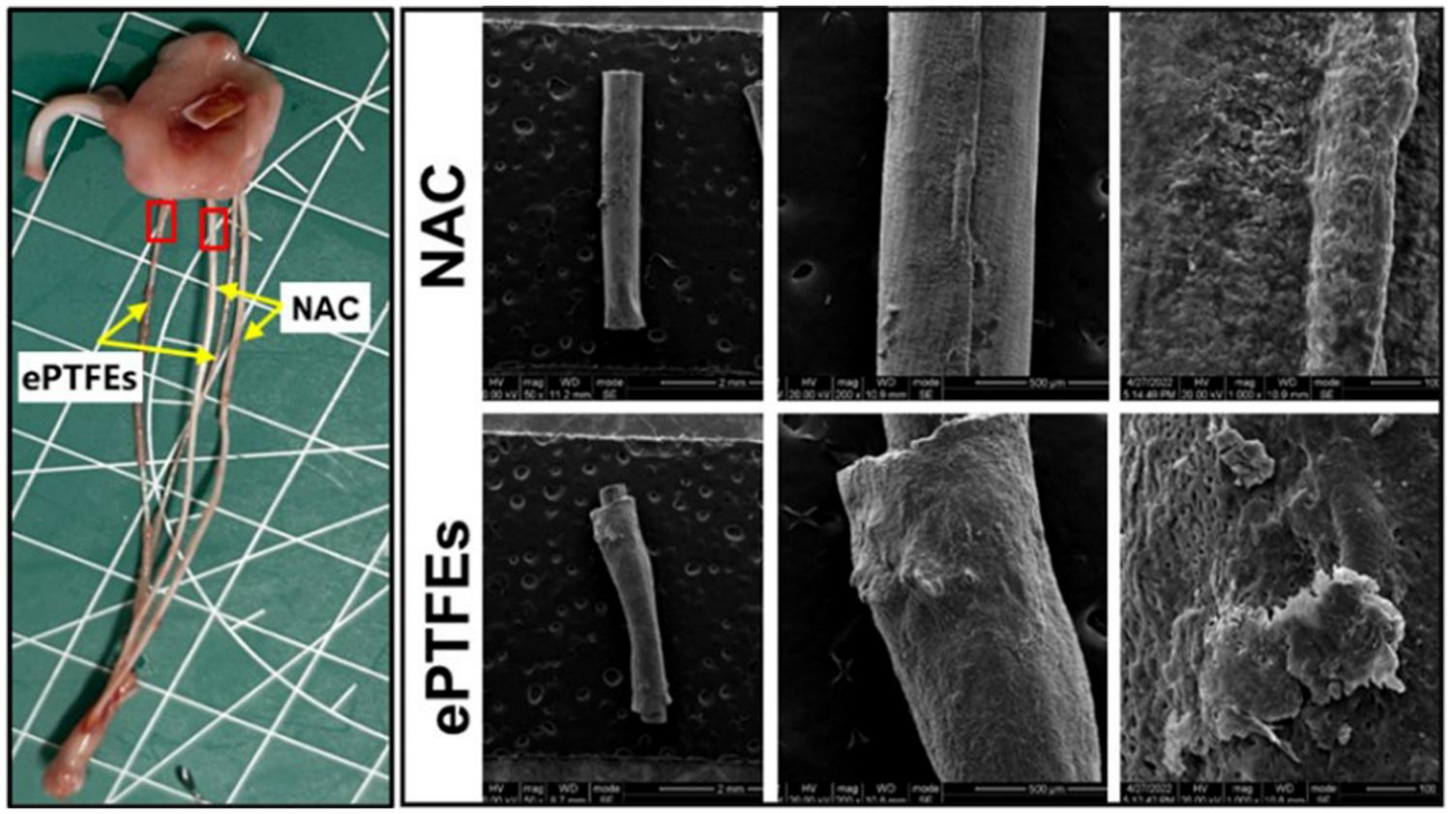
SEM observation of thrombus on the sample surface at 90 days postoperatively.

SEM results (Figure 9) showed that ePTFEs had a thicker deposition of thrombus, forming a continuous shell, and NAC had a smooth, almost thrombus-free and tissue-free surface. The amount of tissue proliferation and the degree of rejection are usually closely related. The results of the subcutaneous implantation experiments (Figure 7) in which the NAC material was implanted with a lesser degree of immune system activation and thus inflammatory response suggest that it has relatively few interactions with the surrounding tissues, which may reduce the degree of tissue rejection and proliferation of the material. In contrast, if the material causes a more severe inflammatory response and rejection, immune cells and inflammatory mediators may accumulate at the implantation site, leading to tissue hyperplasia and scar formation. Therefore, the fact that NAC exhibits a milder inflammatory response may be one of the reasons for less tissue proliferation. This suggests that NAC has better biocompatibility, which helps to minimize adverse biological reactions and improve the success rate of artificial tendon cords. Tissue encapsulation and potential calcification are risk factors for subsequent tendon rupture. Therefore, the mild tissue response characteristics exhibited by NAC indicate its potential to reduce tissue encapsulation, thereby further reducing the potential risk of long-term tendon rupture [23, 24].

#### Histological analysis of implants

**Figure 10.**
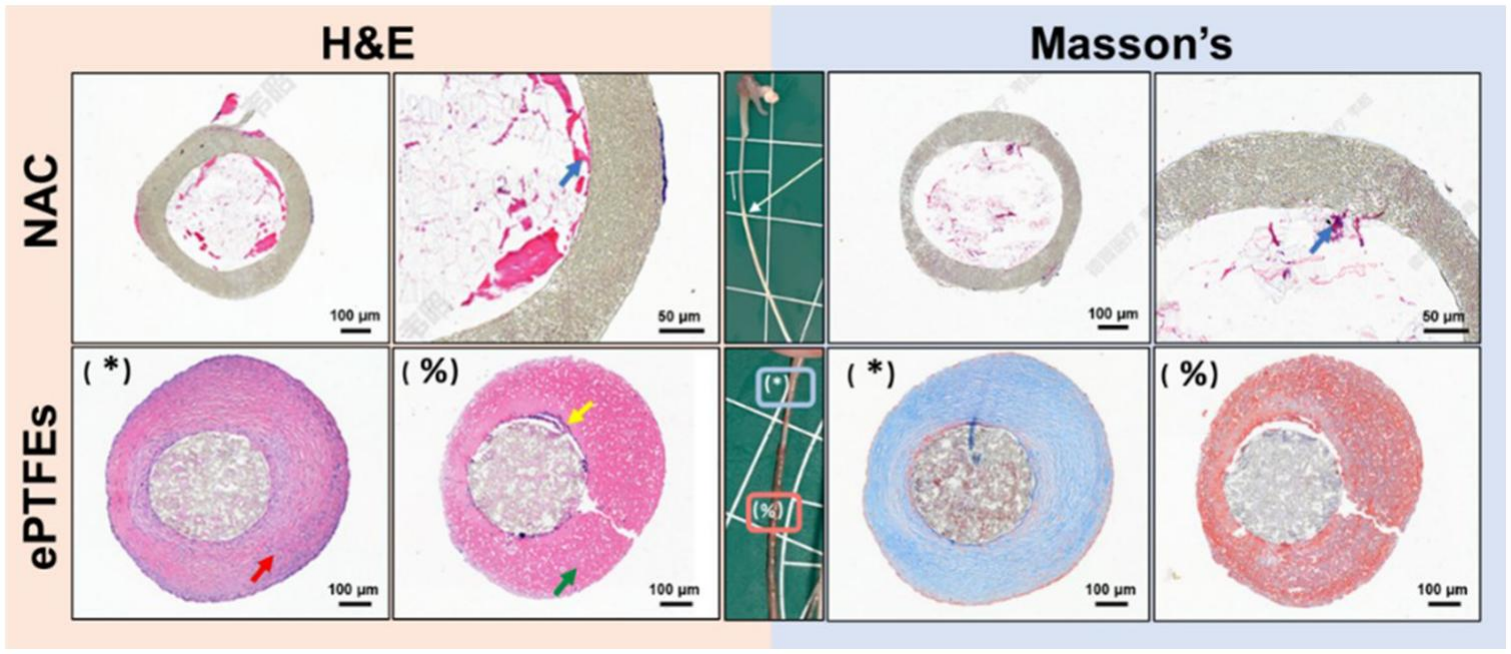
Immunohistochemical staining of neoplastic tissue around implanted cords 90 days after implantation by staining with H&E and Masson’s.

H&E section staining (Figure 10) showed predominantly fibrous tissue formed on the surface near the anchoring area of the ePTFE suture (Red arrow: fibrous tissue; yellow arrow: suspected endothelial cells; green arrow: thrombus; blue arrow: plasma; blue box: fibrous tissue growth; pink box: thrombus encapsulation.), suspected to be formed after mechanization of the thrombus, and the tissue attached midway on the surface of the suture was predominantly thrombus. The surface of the NAC was relatively clean with less coverage of fibrous tissue, with only trace amounts of plasma inside, suggesting that blood could penetrate the interior of the new suture line through the ePTFE cannula. Masson’s stain showed that the main component of the fibrous tissue overlying the ePTFE sutures was collagen, and the overlying thrombus was predominantly fibrin.

These staining results provided insights into the tissue responses and composition surrounding the ePTFE sutures and NAC surfaces. Overall, the ePTFE sutures surfaces somehow triggered tissue reactions, including fibrous tissue and thrombus formation. In contrast, the NAC surface showed little tissue coverage and thrombus formation, possibly indicating that the NAC material was more biocompatible in mitral valve artificial cords and may reduce the risk of adhesion to surrounding tissues. These results have important implications for evaluating the biocompatibility and long-term outcomes of artificial chordae.

## Discussion

Although the evaluations highlighted the advantages of NAC, such as durability and good biocompatibility, and provided preliminary evidence of its safety for use in artificial chordae. However, it was important to acknowledge the limitations of this study. At first, although this study indicated a potential reduction in thrombosis, further research was needed to thoroughly evaluate the compatibility of this material with biological tissues and its potential for adverse reactions. Since the study was limited to preclinical research, it was necessary to conduct further studies using animal models with MR or even subsequent clinical trials to validate the effectiveness and safety of the double-layer artificial chordae in human patients, especially in comparative analyses with other available materials to evaluate its actual clinical benefits.

Furthermore, the study provided initial insights into the durability of the double-layer artificial chordae, but long-term follow-up studies were required to assess its performance over extended periods, considering factors such as cyclic loading, calcification and potential material degradation. Also, it would be essential to consider the cost-benefit analysis and feasibility of manufacturing the tendon cords in large-scale production.

## Conclusion

In the study, a novel double-layer design and fabrication method for high-strength artificial chordae with an anti-adhesive surface was introduced. The incorporation of this design significantly enhanced the mechanical strength of the artificial chordae, improving resistance to high tensile loads and reducing the risk of rupture. The hydrophobic surface layer of NAC effectively mitigated tissue adhesion, thereby enhancing the long-term functionality of the device. Moreover, *in vivo* studies demonstrated excellent biocompatibility, suggesting its potential for clinical applications. These findings highlighted the enhanced durability and functionality of the device, positioning it as a promising solution to address the limitations associated with existing artificial chordae materials. However, further research was required to validate the effectiveness and long-term durability of NAC in human patients. The ultimate objective was to apply the double-layer artificial chordae in clinical practice, offering enhanced treatment options and benefiting patients with heart valve disease.

## Supplementary Material

rbae076_Supplementary_Data
