# Supplementary material for: Design and performance of double-layered artificial chordae
Source: Regen Biomater. 2024 Jun 27;11:rbae076. doi: 10.1093/rb/rbae076 (PMC11269677; doi:10.1093/rb/rbae076)
Supplement: rbae076_Supplementary_Data [file rbae076_supplementary_data.docx]

**Supporting Information**

**Table S1.** Characteristics of the ruptured ePTFE

| Case | Place | Number of reconstructed ePTFE | Number of ruptured ePTFE | Types of ePTFE | Duration between 1^st^ op. and re-do op.(month) | Ruptured point |
| --- | --- | --- | --- | --- | --- | --- |
| 1 | PML | 4 | 2 | CV5 | 201 | Near leaflet |
| 2 | PML | 4 | 2 | CV5 | 44 | middle |
| 3 | PML | 4 | 1 | CV5 | 131 | Near leaflet |
| 4 | PML | 8 | 2 | CV5 | 109 | middle |
| 5 | PML | 4 | 2 | CV5 | 88 | middle |
| 6 | AML | 8 | 6 | CV5 | 66 | middle |
| 7 | AML | 4 | 1 | CV4 | 78 | middle |

op.:operation, PML: posterial mitral leaflet, AML: anterial mitral leaflet.


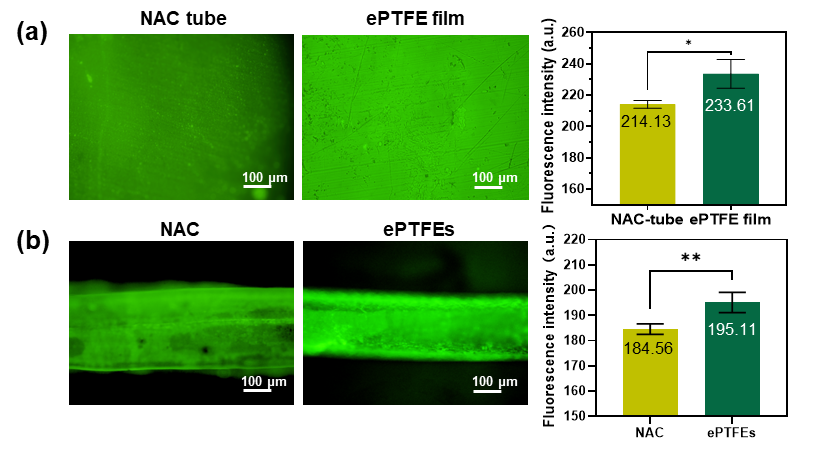


**Figure S1.** (a) Fluorescence photographs and intensity of NAC tube and ePTFE film co-incubated with FITC-BSA. (n=3) (b) Fluorescence photographs and intensity of NAC and ePTFEs co-incubated with FITC-BSA. (n=4). Note: *p < 0.05, ** p < 0.01.


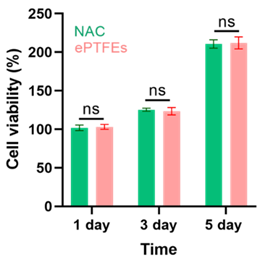


**Figure S2.** Cell viability of HUVECs on NAC and ePTFEs after incubation for 1, 3, 5 days at 37 ℃. (n=6)

**
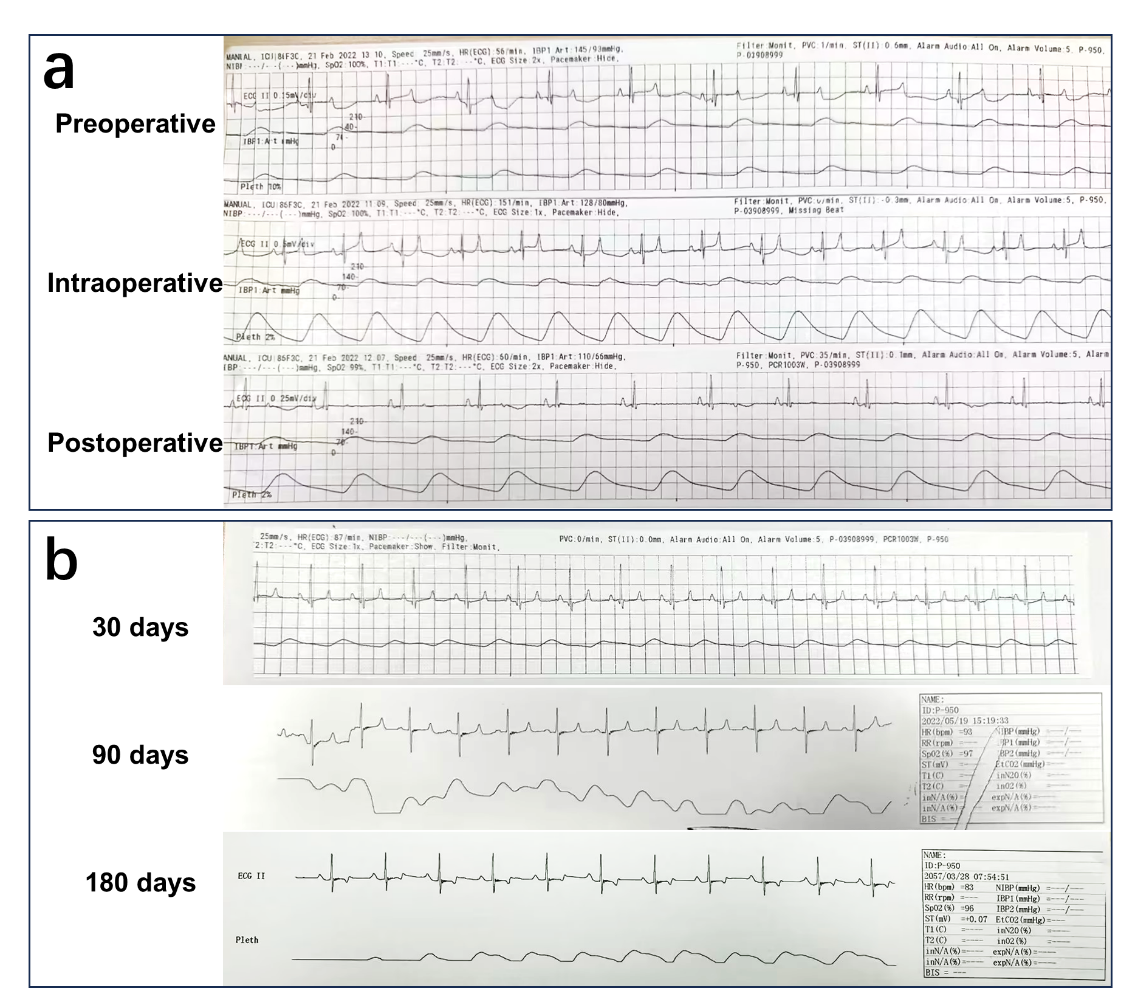
**

**Figure S3.** (a) Electrocardiogram monitoring during surgery. (b) Electrocardiogram monitoring during follow-up.
